# Visible-Light Active Sulfur-Doped Titania Nanoparticles Immobilized on a Silica Matrix: Synthesis, Characterization and Photocatalytic Degradation of Pollutants

**DOI:** 10.3390/nano11102543

**Published:** 2021-09-28

**Authors:** Theodora Kalampaliki, Sofia P. Makri, Evanthia Papadaki, Alexios Grigoropoulos, Alexandros Zoikis Karathanasis, Ioanna Deligkiozi

**Affiliations:** Creative Nano PC, 4 Leventi Street, Peristeri, 12132 Athens, Greece; tkalampa@gmail.com (T.K.); s.makri@creativenano.gr (S.P.M.); e.papadaki@creativenano.gr (E.P.); a.grigoropoulos@creativenano.gr (A.G.); a.karathanasis@creativenano.gr (A.Z.K.)

**Keywords:** photocatalysis, S-doping, TiO_2_ nanoparticles, SiO_2_, MO degradation, NO_x_ oxidation, safe by design

## Abstract

The photocatalytic oxidation (PCO) of pollutants using TiO_2_-based materials can significantly improve indoor air quality (IAQ), which in turn, has a significant impact on human health and life expectancy. TiO_2_-based nanoparticles (NPs) are widely used as part of building materials to function as photocatalysts in PCO. In this work, a series of sulfur-doped TiO_2_ NPs immobilized on a silica matrix were synthesized by combining a sol-gel process with ball milling. The samples were structurally characterized by X-ray diffraction (XRD), UV-Vis diffuse reflectance spectroscopy (DRS), Fourier-transform infrared spectroscopy (FT-IR) and N_2_ adsorption-desorption isotherms. Furthermore, the morphological characteristics were determined by dynamic light scattering (DLS), scanning electron microscopy (SEM) and transmission electron microscopy (TEM). The photocatalytic activity of the as prepared S-doped TiO_2_/SiO_2_ NPs in the degradation of liquid and air pollutants under visible-light irradiation was investigated. Our results show that sulfur is an effective dopant for activating TiO_2_/SiO_2_ photocatalysts under visible-light irradiation. Silica constitutes a “safe-by-design” approach and inhibits the aggregation of NPs during synthesis. The most efficient photocatalyst afforded 79% removal of methyl orange (5 h), 26% removal of acetaldehyde (1 h) and 12% oxidation of NO (1 h).

## 1. Introduction

The levels of air pollutants such as nitrogen oxides (NO_x_), volatile organic compounds (VOCs) and carbon monoxide (CO) in indoor environments can be significantly higher compared with the levels outdoors due to the contribution of indoor sources such as building materials, office equipment, consumer products and combustion by-products [1]. The photocatalytic degradation of these pollutants by nanoparticles (NPs), integrated into building materials and capable of photocatalytically oxidizing these toxic compounds to non-hazardous products, will greatly improve indoor air quality (IAQ). To this end, metal oxide semiconductors such as TiO_2_ [2,3,4,5] and ZnO [6,7] are still considered as the most efficient photocatalysts by virtue of their high chemical stability, low toxicity and low cost.

**Scheme 1:**
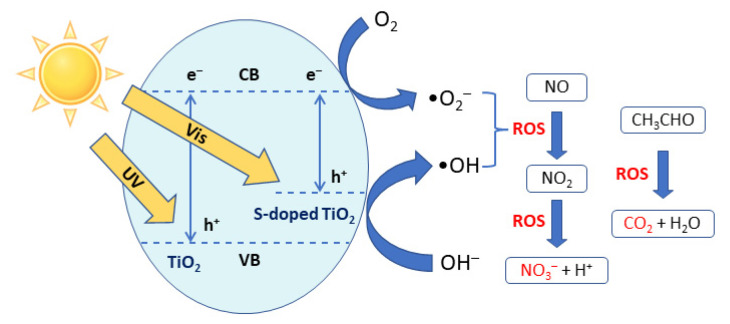
Mechanism for the photocatalytic oxidation of pollutants over TiO_2_-based catalysts.

TiO_2_ exists in three different crystalline phases, i.e., anatase, rutile and brookite. In general, anatase shows better photocatalytic activity; however, anatase and brookite are metastable phases that transform to rutile at higher temperatures (500–600 °C). The well-documented photocatalytic oxidation (PCO) mechanism of TiO_2_ is illustrated in Scheme 1. Light absorption by TiO_2_ can generate electron-hole pairs. The photogenerated electrons can reduce molecular oxygen to superoxide radical anions (•O_2_^−^) or hydrogen peroxide (H_2_O_2_), whereas the corresponding holes can oxidize oxygen-containing surface species such as adsorbed water molecules or Ti-bound hydroxyl groups to hydroxyl radicals (•OH). These highly reactive oxygen species (ROS) can readily decompose common indoor pollutants such as NO_x_ and acetaldehyde [8].

However, TiO_2_ is a semiconductor with a relatively large band gap between 3.0 (pure rutile phase) and 3.2 eV (pure anatase phase). As a result, pure TiO_2_ can only absorb the UV fraction of sunlight which accounts for less than 5% of the solar spectrum. This poses significant limitations, particularly in indoor applications where the intensity of UV radiation is lower. Moreover, the relatively faster charge recombination of electron-hole pairs in pure TiO_2_ photocatalysts, before any type of interaction with the adsorbed species can take place, may further decrease the photocatalytic activity [9,10].

Current challenges in the field aim at the development of novel TiO_2_-based photocatalysts that can operate under visible light radiation and exhibit extended charge separation. Narrowing of the band gap and prolonged charge separation can be achieved via doping of TiO_2_ with metallic and non-metallic species, co-doping with more than one element and coupling of TiO_2_ with other semiconductors [11,12,13,14].

The non-metal doping of TiO_2_ is considered as one of the most efficient methods to increase TiO_2_ photocatalytic activity under visible light. Doping of TiO_2_ with non-metals such as carbon, nitrogen and sulfur can shift the absorption band of TiO_2_ toward the visible region, due to either the generation of new occupied energy levels above the valence band or the formation of oxygen vacancies [15,16,17,18,19]. Nitrogen is the most widely used non-metal dopant due to its small ionization energy and comparable size with oxygen. Nitrogen enters the TiO_2_ lattice as an anion, either occupying an interstitial position or replacing an oxide anion. By contrast, sulfur can be incorporated either as an anion or a cation. Anionic sulfur doping involves the incorporation of sulfide (S^2−^) anions into the lattice, as originally shown by Umebayashi et al. [20]. Cationic doping proceeds via the replacement of Ti^4+^ cations in the TiO_2_ lattice by S^4+^ or S^6+^ cations. The larger radius of sulfide compared with oxide anions makes anionic doping less thermodynamically favored than cationic doping [21,22,23,24,25,26,27,28,29,30].

Ohno et al. were the first to report the cationic S-doping of TiO_2_ using thiourea as the sulfur source [31]. Importantly, S-doped TiO_2_ exhibited stronger visible light absorption compared to C- and N-doped TiO_2_. Lie et al. investigated the effect of the thiourea-to-titanium nominal ratio on the photocatalytic phenyl degradation over S-doped TiO_2_ NPs [22]. A higher amount of S-doping hindered the anatase-to-rutile transformation at higher temperatures and enhanced visible light absorption. However, an upper limit of S-doping was identified above which the photocatalytic activity diminished. The authors concluded that higher levels of S-doping elevated the newly generated energy states to the point where they acted as charge recombination centers. Likewise, an optimal sulfur-to-titanium nominal ratio was observed in the photocatalytic phenol degradation over S-doped TiO_2_ NPs bearing sulfate surface groups [24].

The employment of a supporting matrix with high mechanical and thermal strength, high surface area and available anchoring sites for TiO_2_ NPs could promote photocatalytic activity. To this end, pristine or doped TiO_2_ NPs have been immobilized on metal oxides, carbon nanotubes, reduced graphene oxide, glassy substrates, polymeric surfaces and silica [32,33,34]. Previous works have shown that particularly the employment of silica (SiO_2_) as the support increased the specific surface area of the catalyst, restricted the agglomeration of TiO_2_ NPs and suppressed the anatase-to-rutile phase transformation during synthesis [35,36,37,38,39,40]. The use of silica is also regarded as a “safe-by-design” approach to prevent the release of TiO_2_ NPs into the environment due to strong Si–O–Ti interactions [41,42,43,44,45,46,47].

Nevertheless, to the best of our knowledge, there is only one literature example in which S-doped TiO_2_ NPs were immobilized on silica. Specifically, Chen et al. reported in 2019 the synthesis of SiO_2_-supported cationic S-doped TiO_2_ NPs via the co-hydrolysis of Si- and Ti-based precursors in the presence of thiourea as the sulfur source. The synthesized catalyst was active in the photocatalytic degradation of phenol under visible light irradiation.

In this work, we present a novel and safe-by-design process that combines sol-gel synthesis and ball-milling to produce a series of S-doped TiO_2_/SiO_2_ photocatalysts. The effect of sulfur doping on the photocatalytic properties was probed by varying the nominal sulfur-to-titanium ratio. Generally, the sol-gel synthesis enables control of the reaction parameters and affords nanosized crystalline powders (nanopowders) of high purity and stability [48], whereas ball-milling before calcination leads to smaller NPs with a more uniform particle size distribution [49].

The synthesized photocatalysts were characterized via powder X-ray diffraction (PXRD), scanning electron microscopy (SEM), energy dispersive X-ray spectroscopy (EDS), transmission electron microscopy (TEM), selected area electron diffraction (SAED), dynamic light scattering (DLS), Brunauer-Emmett-Teller (BET) surface area analysis, diffuse reflectance (DRS) and Fourier-transform infrared (FTIR) spectroscopy. The photocatalytic activity in the degradation of methyl orange, acetaldehyde and NO_x_ was investigated. Our results show that S-doped TiO_2_ NPs entrained in a SiO_2_ matrix exhibited substantial activity in the photocatalytic degradation of liquid and air pollutants under visible-light irradiation.

## 2. Experimental Section

### 2.1. Reagents

The following analytical-grade reagents were used as received: titanium(IV) tetraisopropoxide (TTIP, 97%, Sigma-Aldrich, St. Louis, MO, USA) as a precursor of TiO_2_, tetraethyl orthosilicate (TEOS, >99%, Sigma-Aldrich, St. Louis, MO, USA) as a precursor of SiO_2_, thiourea (CH_4_N_2_S, >99%, Penta, Prague, Czech Republic) as the sulfur source and methyl orange (MO, >85%, AppliChem GmbH, Darmstadt, Germany). Absolute ethanol (EtOH, 97%) and deionized water were used as the solvents.

### 2.2. Materials Synthesis and Characterization

In a typical sol-gel synthesis, TEOS (SiO_2_ precursor, 0.30 mL, 1 mmol) was added to an ethanol-water mixture (8 mL EtOH–37 mL H_2_O). The mixture was vigorously stirred for 1 h at room temperature and then TTIP (TiO_2_ precursor, 7.1 mL, 0.024 mol) was added dropwise. This addition sequence was necessary to obtain a uniform sol-gel mixture since TTIP is far more reactive than TEOS [50]. Stirring was continued for 1 h and then thiourea (3.65 g, 0.048 mol) was added. After stirring for 3 h, the mixture was transferred into a crystallization dish and dried at 80 °C overnight. Subsequently, the sample was ground for 30 min at 350 rpm by planetary ball-milling, calcined at 500 °C for 2 h in static air and finally ground again as above. The synthesized sample was denoted as S(2)-TiO_2_/SiO_2_. Two more samples were synthesized using a higher nominal S/Ti ratio. Specifically, 8.2 g (0.108 mol) or 12.79 g (0.168 mol) of thiourea was added to produce S(4)-TiO_2_/SiO_2_ or S(6)-TiO_2_/SiO_2_, respectively. For the sake of comparison, pure TiO_2_, undoped TiO_2_/SiO_2_ and unsupported S-doped TiO_2_ samples were also synthesized following the same synthetic protocol without adding thiourea or TEOS.

The PXRD patterns were collected on a Bruker D8 Advance (Karlsruhe, Germany) diffractometer with Cu K*α* radiation (*λ* = 0.15418 nm). The accelerating voltage and applied current were 40 kV and 40 mA, respectively. Profiles were measured in the 20° < 2*θ* < 80° range with a step of 0.04°/2 s. The crystalline phases were identified with reference to the PDF cards of the International Centre for Diffraction Data (ICDD). The average crystallite size of the anatase phase was determined from the intensity of the main (101) reflection using the Scherrer equation, as follows:

(1) $$d\left(\mathrm{nm}\right)=\frac{0.89\times \lambda }{\beta \times \;\cos\left(\theta \right)}$$

where *λ* (nm) is the X-ray wavelength, *β* (rad) is the full width at half the maximum of the peak intensity and *θ* is the respective Bragg angle. Particle morphology was investigated by SEM on a Jeol 6380 LV instrument (Tokyo, Japan) equipped with an Oxford Instruments INCA EDS system (Abingdon, UK). The electron beam accelerating voltages were between 15–20 kV. To improve the surface conductivity of the samples, standard gold deposition was applied through vacuum evaporation. UV-Vis spectra in solution and diffuse reflectance spectra in the solid state were measured between 350–800 nm using an Agilent Carry 60 spectrometer (Santa Clara, CA, USA). The DRS measurements were performed using a Harrick VideoBarrelino DRA fiber optic coupler (Pleasantville, NY, USA). The band gap was calculated using the Kubelka-Munk (K-M) model by plotting [F(R) × E]^1/2^ vs. E (eV), where F(R) = (1 − R)^2^/2R is the K-M function and R is the reflectance of the materials.

N_2_ adsorption-desorption isotherms at 77 K were measured on a Quantachrome NOVA 1200 gas analyzer (Boynton Beach, FL, USA). Samples were degassed at 150 °C for 3 h before measurement. The specific surface area (*SSA_exp_*) of the samples was determined via the BET model using the multipoint method of the Quantochrome NovaWin2 software (version 2.2). The pore size distribution was obtained by applying Barret-Joyner-Halenda (BJH) analysis for the desorption part of the isotherm. The theoretical *SSA* of the TiO_2_ nanoparticles (*SSA_calc_*) was calculated based on the average particle diameter, estimated by XRD (*d_XRD_*) and the weighted density of anatase (*ρ_A_* = 3.84 gr cm^−3^), assuming spherical and non-agglomerated particles, as follows:

(2) $$SSA_{calc}=\frac{6}{\left(\rho_{A}\times d_{XRD}\right)}$$

Particle size distribution was also measured via DLS using an Anton Paar Litesizer 500 particle size analyzer (Graz, Austria). Particles were suspended in water via ultrasonication for 1 min prior to measurement. The FTIR spectra of the synthesized samples were measured on a Brucker Tension 27 FTIR spectrometer (Karlsruhe, Germany) equipped with a diamond ATR accessory at a spectral resolution of 4 cm^−1^ in the 4000–600 cm^−1^ range. IR spectra were analyzed with the Bruker OPUS software (version 5.2).

TEM images were collected on a Jeol 2100 HR (Tokyo, Japan) microscope operating at 200 kV. The TEM samples were prepared as follows: a small amount of the photocatalyst was dispersed in ethanol and the suspension was sonicated for 10 min. Afterwards, a single drop of the suspension was placed on a carbon-coated grid and was allowed to dry at ambient temperature. SAED patterns were analyzed with ImageJ software (LOCI, Madison, WI, USA).

### 2.3. Photocatalytic Evaluation Methods

The synthesized powders were evaluated as photocatalysts in the decomposition of pollutants in an aqueous medium using methyl orange (MO) as a model compound, based on the relevant ISO 10678:2010 standard procedure [51]. In a typical experiment, 50 mg of the powder sample was combined with 150 mL of an aqueous solution of MO (2 mg L^−1^). The mixture was sonicated for 15 min, stirred in the dark for 40 min and afterwards irradiated with visible light for 5 h using 4 parallel Daylight 18 W lamps and a UV cut-off filter (99% UV cut-off capability). Aliquots (3 × 1 mL) were collected at 60 min intervals and centrifuged. The concentration of MO in the supernatant was determined by UV-Vis spectroscopy. A control experiment was also run during which an identical system was kept in the dark for 5 h in order to correct for the amount of MO adsorbed on the surface of the photocatalyst.

Regarding the PCO of air pollutants, nitric oxide (NO) and acetaldehyde (CH_3_CHO) were selected as representative airborne inorganic and organic pollutants, respectively. The photocatalytic activity of all samples was investigated following the ISO 22197–1:2007 and ISO 22197-2:2011 standard procedures [52,53]. A detailed description of the experimental set up and parameters are provided in the literature [54]. A constant flow rate of 3 L min^−1^ and a relative humidity of 50% were maintained during all the experiments. Visible light irradiation (~7000 lux) was applied for 60 min. A short dark period of 5 min preceded irradiation to allow the mixture to reach equilibrium and correct for adsorption of pollutants on the photocatalyst.

The concentration of acetaldehyde was determined by a Shimadzu Tracera High Sensitivity GC 2010 (Kyoto, Japan). The concentrations of NO, NO_2_ and NO_x_ (NO_x_ = NO + NO_2_ combined concentration) were determined by a HORIBA 370 analyzer (Kyoto, Japan) by integrating the respective peak area over time. The activity of the photocatalysts was calculated using Equations (3)–(5):

(3) $$Effectiveness\;\left(\mathrm{NO}\right)=\frac{\int_{t0}^{t1}\left(\mathrm{NO}\;\mathrm{initial}-\mathrm{NO}\;\mathrm{measured}\right)dt\;}{\left(\mathrm{NO}\;\mathrm{initial}\;\right)\times T}$$

(4) $$Effectiveness\;\left({\mathrm{NO}}_{2}\right)=\frac{\int_{t0}^{t1}\left({\mathrm{NO}}_{2}\;\mathrm{measured}-{\mathrm{NO}}_{2}\;\mathrm{initial}\right)dt\;}{\left({\mathrm{NO}}_{2}\;\mathrm{initial}\;\right)\times T}$$

(5) $$Effectiveness\;\left({\mathrm{NO}}_{x}\right)=\frac{\int_{t0}^{t1}\left({\mathrm{NO}}_{x}\;\mathrm{initial}-{\mathrm{NO}}_{x}\;\mathrm{measured}\right)dt\;}{\left({\mathrm{NO}}_{x}\;\mathrm{initial}\right)\times T}$$

where *T* = *t*_1_ − *t*_0_ corresponds to the period of visible irradiation, during which the lamp was switch on at *t* = *t*_0_ and switched off at *t* = *t*_1_. The effectiveness of each photocatalyst per compound corresponds to the change in the compound’s concentration at the end of the irradiation period, expressed as a percentage.

## 3. Results and Discussion

### 3.1. Powder XRD and Porosity Analysis

**Figure 1:**
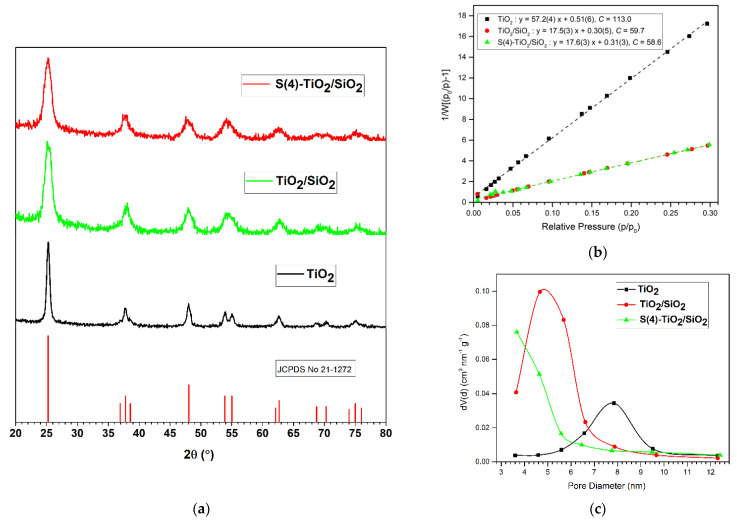
(**a**) Powder XRD patterns, (**b**) BET plots and (**c**) BJH pore size distribution of TiO_2_, TiO_2_/SiO_2_ and S(4)-TiO_2_/SiO_2_ samples.

The powder XRD patterns of the TiO_2_, TiO_2_/SiO_2_ and S(4)-TiO_2_/SiO_2_ samples (Figure 1a) showed diffractions peaks characteristic of anatase TiO_2_ (JCPDS 21-1272), whereas peaks corresponding to the rutile or brookite phases were not observed. Therefore, powder XRD indicated that the synthetic protocol adopted herein led to the formation of a single crystalline TiO_2_ phase, anatase. The average crystallite size (d_XRD_) was determined using the Scherrer equation (Equation (1)) on the (101) main diffraction peak of anatase at 2*θ* = 25.28°. All the diffraction peaks in the XRD patterns of TiO_2_/SiO_2_ and S(4)-TiO_2_/SiO_2_ were broader compared with those of pure TiO_2_, suggesting that the growth of the TiO_2_ particles was restricted when SiO_2_ was introduced. This was reflected by the average crystallite size which was significantly reduced from 20 nm for pure TiO_2_, to 7.6 nm for TiO_2_/SiO_2_ and 6.7 nm for S(4)-TiO_2_/SiO_2_ (Table 1). Therefore, S-doping resulted in even smaller particles, probably due to the substitution of Ti^4+^ by S^6+^ and S^4+^ cations, which can further restrict the growth of TiO_2_ particles [28].

These observations were supported by N_2_ adsorption-desorption measurements. The BET plots and the BJH pore size distributions for TiO_2_, TiO_2_/SiO_2_ and S(4)-TiO_2_/SiO_2_ are presented in Figure 1b,c, respectively. The BET surface area and the pore volume increased from *SSA_exp_* = 60 m^2^ g^−1^ and V_P_ = 0.198 cm^3^ g^−1^ for TiO_2_ to *SSA_exp_* = 195 m^2^ g^−1^ and V_P_ = 0.315 cm^3^ g^−1^ for TiO_2_/SiO_2_ (Table 1), in good agreement with the calculated values (*SSA_calc_*).

The higher porosity was accompanied by a decrease in pore diameter from 7.87 nm for TiO_2_ to 4.67 nm for TiO_2_/SiO_2_. This, in turn, suggested that the d_XRD_ values calculated from the (101) diffraction peak of the respective powder XRD patterns nearly represented the true size of the TiO_2_ nanoparticles, as verified by TEM images (see below Section 3.4). S-doping of TiO_2_/SiO_2_ had no effect on the BET surface area (*SSA_exp_* = 195 m^2^ g^−1^) compared with TiO_2_/SiO_2_. However, the pore volume (V_P_ = 0.277 cm^3^ g^−1^) and pore diameter (3.67 nm) decreased compared with undoped TiO_2_/SiO_2_. This indicated that S-doping resulted in partial pore blocking, in agreement with literature reports [22,24,55].

### 3.2. FTIR Spectroscopy

**Figure 2:**
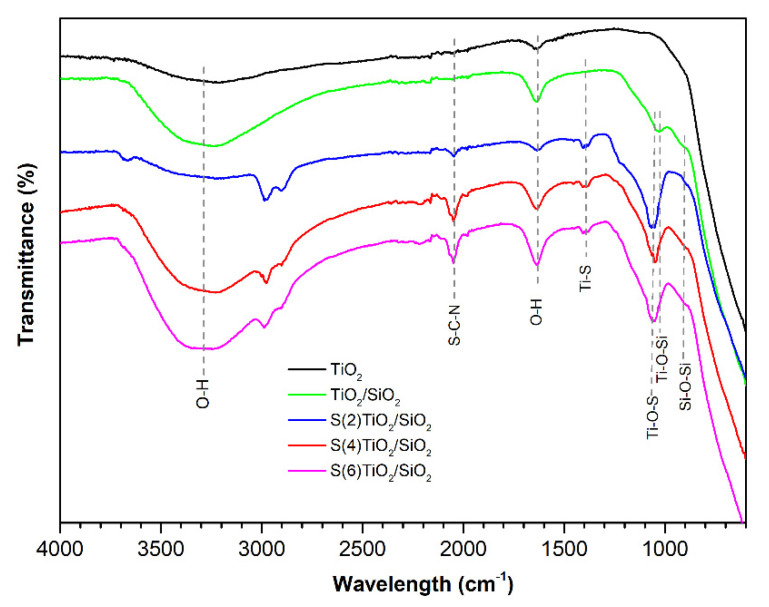
FTIR spectra of (as prepared) TiO_2_, TiO_2_/SiO_2_ and S-doped TiO_2_/SiO_2_ samples.

FTIR analysis was performed to identify the nature of the chemical bonds in the synthesized samples. The FTIR spectra of TiO_2_, TiO_2_/SiO_2_ and S(x)-TiO_2_/SiO_2_ (x = 2, 4, 6) are presented in Figure 2. The peaks at 3300–3500 cm^−1^ and 1640 cm^−1^ were observed in all spectra. These peaks were assigned to the stretching and bending vibrations of adsorbed water molecules and surface hydroxyl groups, as previously reported [24]. Two more peaks at 1028 cm^−1^ and 907 cm^−1^ were observed in the FTIR spectrum of undoped TiO_2_/SiO_2_, which have previously been assigned to the stretching vibration of the Si–O–Si and Ti–O–Si groups, respectively [30,56]. The detection of the Ti–O–Si vibration at 907 cm^−1^ was consistent with the incorporation of Ti into the silica framework in undoped TiO_2_/SiO_2_.

With respect to the S(x)-TiO_2_/SiO_2_ samples, the intensity of the *v*(O–H) peaks at 3300–3500 cm^−1^ and *δ*(O–H) at 1640 cm^−1^ increased with the increasing nominal S/Ti ratio, indicative of a gradually greater number of surface hydroxyl groups at higher levels of S-doping [30]. Moreover, three peaks were observed at 2050, 1400 and 1060 cm^−1^, which were absent in the FTIR spectrum of undoped TiO_2_/SiO_2_. Based on previous reports on S-doped TiO_2_ [23,24,30,57], the higher energy peak at 2050 cm^−1^ was assigned to the C–N stretching vibration of the isothiocyanate group –NCS, whereas the two lower energy peaks at 1400 and 1060 cm^−1^ were attributed to Ti–S and Ti–O–S stretching vibrations. It should be underlined that the intensity of the above peaks increased when larger quantities of thiourea were added in the reaction mixture. These observations were consistent with the presence of Ti–NCS and Ti–S bonds in the synthesized photocatalysts.

### 3.3. UV–Vis Diffused Reflectance Spectroscopy: Band Gap Analysis

**Figure 3:**
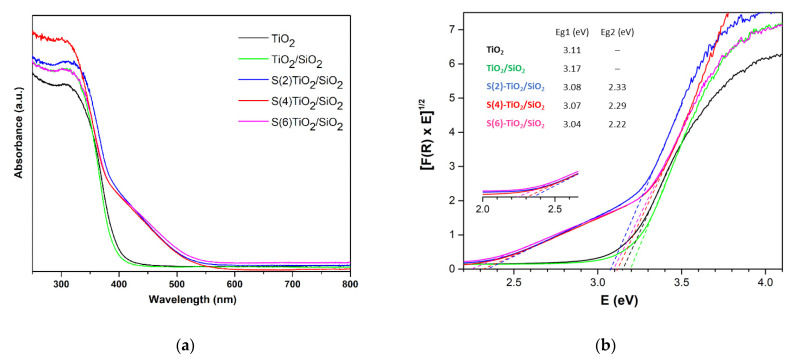
(**a**) Diffuse reflectance spectra and (**b**) Tauc plots for TiO_2_, TiO_2_/SiO_2_ and S-doped TiO_2_/SiO_2_ samples.

The DR spectra for all samples are presented in Figure 3a. As expected, the colorless semiconductors TiO_2_ and TiO_2_/SiO_2_ showed a single absorption edge in the UV region of the spectrum. By contrast, a second absorption edge in the visible region was observed for all the S-doped TiO_2_/SiO_2_ nanopowders, suggesting the presence of a localized band above the main valence band of TiO_2_, as previously reported [22,30,58,59]. The respective Tauc plots (Figure 3b), based on the Kubelka-Munk model, revealed that the energy band gap values corresponding to each edge—Eg1 and Eg2, respectively—decreased as the amount of thiourea in the sol-gel mixture increased (Table 2). As a result, the lowest Eg2 value of 2.22 eV was found for S(6)-TiO_2_/SiO_2_, the sample with the higher level of S-doping.

### 3.4. Morphological Analysis

**Figure 4:**
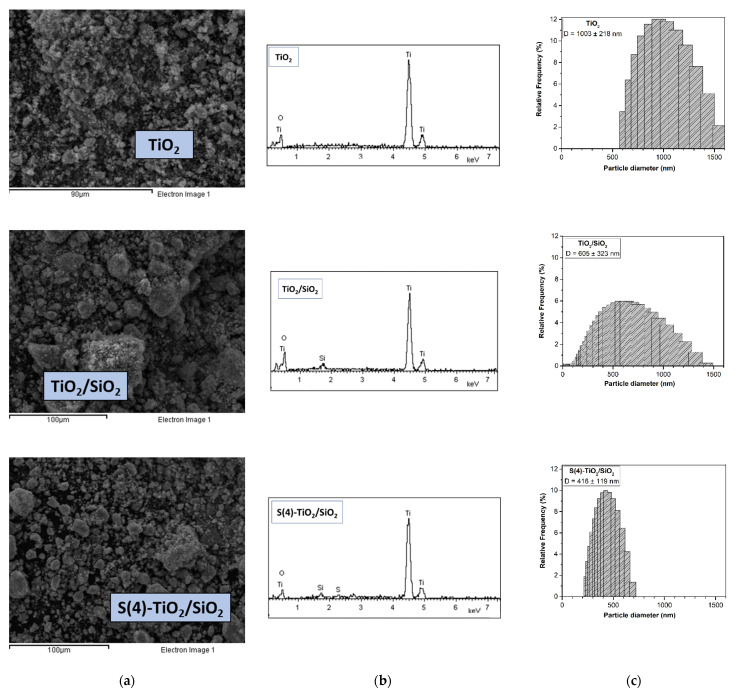
(**a**) SEM images, (**b**) EDS analysis and (**c**) DLS analysis of TiO_2_, TiO_2_/SiO_2_ and S(4)-TiO_2_/SiO_2_ samples.

Figure 4 shows characteristic SEM images, EDS analysis and DLS particle size distribution of TiO_2_, TiO_2_/SiO_2_ and S(4)-TiO_2_/SiO_2_. Aggregates with particle sizes ranging from 400 nm to 40 μm were observed for all samples. However, the S(4)-TiO_2_/SiO_2_ powder had a more porous morphology and the particle size was relatively confined. Importantly, EDS analysis verified the presence of silicon in TiO_2_/SiO_2_, as well as sulfur and silicon in S(4)-TiO_2_/SiO_2_. To probe the effect of silica and S-doping on particle size, DLS measurements were also performed after dispersing the NPs in water. Supporting TiO_2_ on silica led to a decrease in particle size from *d* = 1003 ± 218 nm for TiO_2_ to *d* = 605 ± 323 for TiO_2_/SiO_2_. S-doping led to even smaller particles with a narrow size distribution, i.e., *d* = 416 ± 119 nm for S(4)-TiO_2_/SiO_2_.

**Figure 5:**
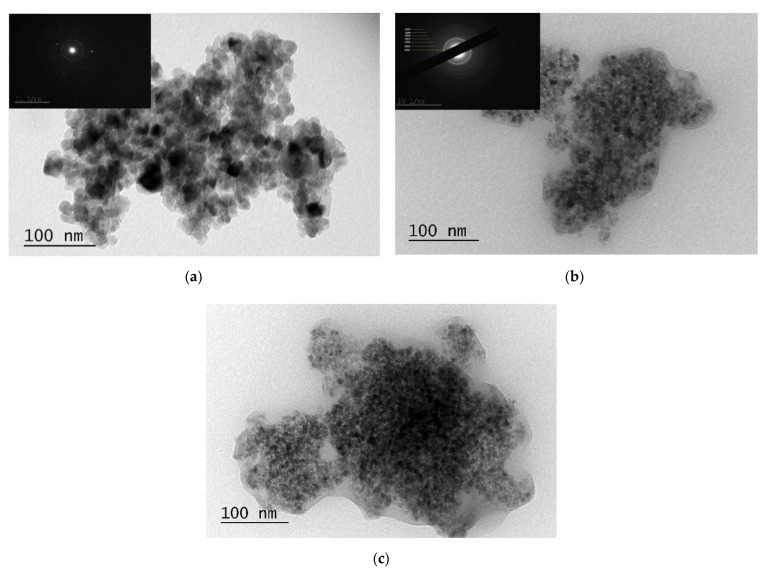
TEM images and SAED patterns (inset) of (**a**) TiO_2_, (**b**) TiO_2_/SiO_2_ and (**c**) S(4)-TiO_2_/SiO_2_ samples.

More detailed morphological and structural characterization of the samples was carried out using TEM (Figure 5). The TEM micrographs of all samples revealed numerous aggregates and ill-defined nanostructures. However, the TiO_2_/SiO_2_ and S(4)-TiO_2_/SiO_2_ samples demonstrated better dispersion and smaller crystallite size compared with pure TiO_2_. Specifically, the crystallite size calculated from the TEM images was approximately 20 nm for pure TiO_2_ (Figure 5a), in accordance with the XRD results. The introduction of SiO_2_ and S-doping resulted in considerably smaller particles, between 5–10 nm. Importantly, the TEM images of the composites TiO_2_/SiO_2_ and S(4)-TiO_2_/SiO_2_ revealed that the TiO_2_ nanoparticles were almost completely surrounded by a silica layer (Figure 5b,c). As has already been reported, the opposite surface charge of TiO_2_ and SiO_2_ could promote the encapsulation of TiO_2_ nanoparticles into the SiO_2_ matrix through electrostatic interactions [43,44]. It is, therefore, likely that encapsulation of TiO_2_ inside the SiO_2_ matrix restricted the accumulation of larger TiO_2_ nanoparticles during the synthesis of the photocatalysts.

Furthermore, SAED patterns showed a sequence of spots for TiO_2_ and rings for undoped TiO_2_/SiO_2_ (inset—Figure 5a,b). The detection of distinct diffraction spots in the case of TiO_2_ suggested a more crystalline sample and a larger crystallite size. On the contrary, the observation of diffraction rings in the case of TiO_2_/SiO_2_ indicated a less crystalline sample and a smaller particle size. Therefore, the TEM images were in accordance with the powder XRD analysis (Section 3.1). Moreover, the interplanar spacings derived from the diffraction rings were *d*(215) = 0.35 nm, *d*(004) = 0.24 nm, *d*(200) = 0.19 nm, *d*(211) = 0.17 nm, *d*(204) = 0.15 nm and *d*(211) = 0.13 nm. These values corresponded to the anatase TiO_2_ crystalline phase (PDF no 21-1272), as also suggested by the powder XRD pattern.

### 3.5. Photocatalytic Evaluation

#### 3.5.1. Liquid Pollutant Degradation

**Figure 6:**
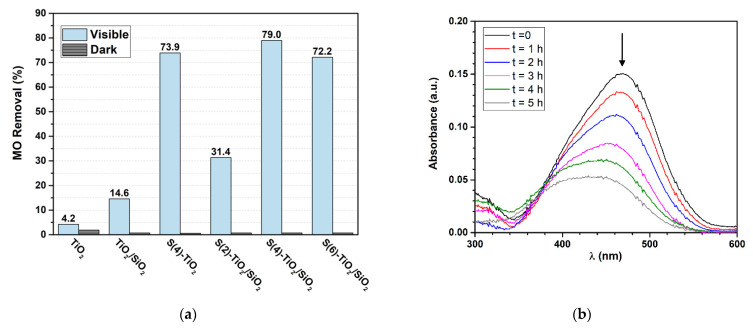
(**a**) MO removal (%) in aqueous media over TiO_2_, TiO_2_/SiO_2_, S(4)-TiO_2_ and S(x)-TiO_2_/SiO_2_ samples under visible light irradiation for 5 h; (**b**) Overlay of absorbance spectra for MO degradation over S(4)-TiO_2_/SiO_2_, collected at 1 h intervals.

The photocatalytic activity of the as-synthesized samples was initially benchmarked against the photocatalytic oxidation of MO under visible light irradiation. The results are presented in Figure 6a alongside the related control experiments, which were carried out in the dark. As expected, pure TiO_2_ showed negligible activity, removing only 4.2% of MO in solution after 5 h. A minor increase of activity was observed for undoped TiO_2_/SiO_2_, which afforded 14.6% MO removal, most likely due to the smaller TiO_2_ particle size and the larger porosity of the TiO_2_/SiO_2_ nanocomposite, as shown above (Table 1).

The S-doped samples showed significantly higher activity. S(4)-TiO_2_/SiO_2_ was the most efficient photocatalyst, affording 79.0% MO removal after 5 h. Figure 6b shows the corresponding UV-Vis absorption spectra, collected at 1 h intervals. The intensity of the characteristic absorption peak of MO at 470 nm rapidly decreased in 5 h. The S(2)-TiO_2_/SiO_2_ and S(6)-TiO_2_/SiO_2_ photocatalysts afforded 31.4% and 72.2% MO removal, respectively. Therefore, an optimal thiourea-to-titanium nominal ratio was required to maximize the photocatalytic activity in MO degradation. As previously reported [22,24,60], lower levels of S-doping limit the ability of the catalyst to operate under visible light, whereas higher levels of S-doping enhance charge recombination. Finally, it should be noted that the unsupported S(4)-TiO_2_ photocatalyst afforded 73.9% MO removal in 5 h. Therefore, the use of silica as a matrix did not significantly improve the photocatalytic activity in MO degradation despite the higher porosity of the SiO_2_-supported samples, most likely due the rather large size of MO, which in turn, slows down its diffusion within the pores.

In summary, S-doping of TiO_2_ resulted in a significantly higher photocatalytic activity in terms of MO degradation under visible light irradiation. This could be attributed to the narrower band gap induced by S-doping (Figure 3b), as well as the higher number of surface hydroxyl groups in the S-doped TiO_2_ samples, as indicated by FTIR spectroscopy (Figure 2) [61].

#### 3.5.2. Air Pollutant Degradation

**Figure 7:**
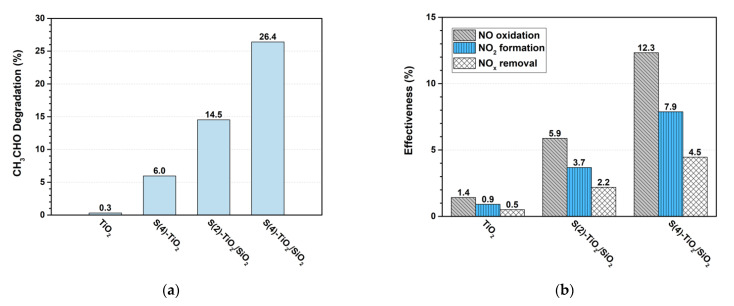
(**a**) Acetaldehyde photocatalytic degradation (%) in air over TiO_2_, S(4)-TiO_2_ and S(x)-TiO_2_/SiO_2_ samples; (**b**) NO_x_ photocatalytic degradation (effectiveness, %) in air over TiO_2_, S(2)-TiO_2_/SiO_2_ and S(4)-TiO_2_/SiO_2_. Conditions: flow rate of 3 L min^−1^, RH of 50% and visible light irradiation (~7000 lux) for 1 h.

The photocatalytic activity of the as-synthesized samples was also evaluated in the photocatalytic degradation of CH_3_CHO and NO(g). Figure 7 presents a comparison of the effectiveness of the photocatalysts, i.e., the change in concentration of each compound, expressed as a percentage. As also observed for MO degradation, the S-doped catalysts were significantly more active under visible light irradiation compared with TiO_2_, which exhibited negligible activity. Specifically, the acetaldehyde concentration was reduced by 6.0% over unsupported S(4)-TiO_2_, by 14.5% over S(2)-TiO_2_/SiO_2_ and by 26.4% over S(4)-TiO_2_/SiO_2_ in 1 h under visible light irradiation (Figure 7a). Therefore, entraining S-doped TiO_2_ NPs in a silica matrix, clearly had a beneficial effect in the case of acetaldehyde degradation, a small molecule that can readily diffuse through the pores.

With respect to the photocatalytic degradation of NO(g) over TiO_2_-based materials, it is widely accepted that NO is initially oxidized to NO_2_(g) and subsequently to NO_3_^−^ by photogenerated hydroxyl (•OH) rather than superoxide (•O^2−^) radicals [62]. Along these lines, Todorova et al. reported that N,S co-doping of TiO_2_ using thiourea as the source had a detrimental effect on the photocatalytic degradation of NO_x_ under visible-light irradiation because the formation of •OH radicals was suppressed [25].

On the contrary, the TiO_2_-based photocatalysts synthesized in this work using thiourea as the source and silica as a supporting matrix were moderately active in NO_x_ decomposition under visible-light irradiation. In particular, the S(2)-TiO_2_/SiO_2_ catalyst afforded 5.9% NO oxidation to NO_2_ and 2.2% combined NO_x_ removal in 1 h. The catalytic activity significantly increased over S(4)-TiO_2_/SiO_2_, which afforded 12.3% NO oxidation and 4.5% total NO_x_ removal in 1 h. Clearly, the higher porosity and the smaller particle size of the photocatalysts associated with the use of silica as a matrix (see Table 1) promoted the adsorption of •OH radicals on the catalytic surface, which in turn, resulted in the enhancement of NO to NO_2_ oxidation. Nevertheless, NO_2_ was only partially further oxidized to NO_3_^−^, resulting in lower efficiency values with respect to total NO_x_ removal (NO + NO_2_). This suggested that NO_3_^−^ products could partially block active sites on the catalytic surface.

## 4. Conclusions

This study demonstrated the feasibility of producing silica-supported S-doped TiO_2_ nanopowders with substantial activity in the photocatalytic degradation of liquid (MO) and air (CH_3_CHO and NO_x_) pollutants under visible light irradiation. S-doping using thiourea as the source shifted the absorption towards the visible region of the spectrum to enable photocatalytic activity under visible light. Moreover, the ‘safe-by-design’ strategy of entraining the S-doped TiO_2_ NPs within a silica layer improved the photocatalytic activity by increasing the surface area, reducing the particle size and facilitating the diffusion of small-sized pollutants toward the catalytically active sites.

## Figures and Tables

**Table 1:** Textural properties of TiO_2,_ TiO_2_/SiO_2_ and S(4)-TiO_2_/SiO_2_ samples.

Sample	*d_XRD_* ^[a]^	*SSA_calc_* ^[b]^	*SSA_exp_* ^[c]^	*V_P_* ^[d]^	Pore Diameter
	(nm)	(m^2^ gr^−1^)	(m^2^ gr^−1^)	(cm^3^ g^−1^)	(nm)
TiO_2_	20	78	60	0.198	7.85
TiO_2_/SiO_2_	7.6	206	195	0.315	4.67
S(4)-TiO_2_/SiO_2_	6.7	233	195	0.277	3.67

^[a]^ Particle size based on powder *XRD*, ^[b]^ Calculated specific surface based on *d_XRD_*, ^[c]^ Experimental specific surface based on BET model, ^[d]^ Pore volume.

**Table 2:** Band gap values (Eg1 and Eg2) of TiO_2_, TiO_2_/SiO_2_ and S(x)-TiO_2_/SiO_2_ samples.

Sample	Eg1	Eg2
	(eV)	(eV)
TiO_2_	3.11	-
TiO_2_/SiO_2_	3.17	-
S(2)-TiO_2_/SiO_2_	3.08	2.33
S(4)-TiO_2_/SiO_2_	3.07	2.29
S(6)-TiO_2_/SiO_2_	3.04	2.22

## Data Availability

Not applicable.
